# Short-Term Inhibition of NOX2 Prevents the Development of Aβ-Induced Pathology in Mice

**DOI:** 10.3390/antiox14060663

**Published:** 2025-05-30

**Authors:** Kristina A. Mukhina, Olga I. Kechko, Alexander A. Osypov, Irina Yu. Petrushanko, Alexander A. Makarov, Vladimir A. Mitkevich, Irina Yu. Popova

**Affiliations:** 1 Engelhardt Institute of Molecular Biology of the Russian Academy of Sciences, 119991 Moscow, Russia; kristina.mukhina@gmail.com (K.A.M.); olga.kechko@gmail.com (O.I.K.); aosypov@gmail.com (A.A.O.); irina-pva@mail.ru (I.Y.P.); aamakarov@eimb.ru (A.A.M.); 2 Institute of Theoretical and Experimental Biophysics of the Russian Academy of Sciences, 142290 Pushchino, Russia; 3 Institute of Higher Nervous Activity and Neurophysiology of the Russian Academy of Sciences, 117485 Moscow, Russia

**Keywords:** Alzheimer’s disease model, oligomeric Aβ, GSK2795039, neuroinflammation, oxidative stress, microglia, aggressive behavior

## Abstract

Alzheimer’s disease (AD) is the most common neurodegenerative disorder, characterized by the formation of neurotoxic beta-amyloid (Aβ) oligomers in the central nervous system. One of the earliest pathological effects of Aβ is the induction of oxidative stress in brain tissue, mediated by NADPH oxidase 2 (NOX2). This study aimed to determine whether short-term inhibition of NOX2 could disrupt the pathological cascade and prevent the development of Aβ-induced pathology. We demonstrated that suppressing NOX2 activity by GSK2795039 during the first three days after intracerebral Aβ administration prevented the development of the pathological process in mice. Two weeks after the induction of Aβ pathology, animals treated with GSK2795039 showed no neuropsychiatric-like behavioral changes, which correlated with the absence of chronic oxidative damage in brain tissue. Moreover, GSK2795039 prevented microglial activation and reduced microglia-associated neuroinflammation. These findings indicate that short-term NOX2 inhibition effectively suppresses the development of Aβ-induced pathology, suggesting that NOX2 is a potential target for treatment and prevention of AD pathology.

## 1. Introduction

Alzheimer’s disease (AD) is a slowly progressive neurodegenerative disorder that leads to cognitive impairment and memory loss. The neuropathological hallmarks of AD include senile plaques composed of extracellular fibrillar beta-amyloid (Aβ) deposits and intracellular neurofibrillary tangles consisting of hyperphosphorylated tau protein [1,2,3]. Although the precise mechanisms underlying AD remain incompletely understood, it is becoming clear that oxidative stress and neuroinflammation are critical factors contributing to the progression of neurodegeneration [4] and the formation of senile plaques [5].

Senile plaques are surrounded by activated microglia, which play a major role in promoting and sustaining a pro-inflammatory environment in the affected brain regions. Microglial cells, the resident macrophages of the central nervous system [6], participate in phagocytosis, cytokine production, and the encapsulation of amyloid plaques [7,8]. Contact with beta-amyloid oligomers triggers persistent microglial activation and leads to the formation of microglial clusters around Aβ deposits that already occur in early stages of the pathology [9,10,11,12]. This interaction is mediated by microglial surface receptors that bind to Aβ [13,14]. The sustained microglial activation resulting from Aβ accumulation leads to chronically elevated production of pro-inflammatory factors and free radicals that are toxic to neurons and oligodendrocytes, while also promoting further Aβ production [15,16,17].

Currently, the suppression of oxidative stress and related neuroinflammation is considered to be one of the primary strategies for developing effective AD treatments [4,18,19,20,21,22,23,24,25]. The therapeutic approach for neurodegenerative diseases has evolved from targeting antioxidants and traditional anti-inflammatory pathways toward modulating upstream mediators, following the limited clinical success of most antioxidant and nonsteroidal anti-inflammatory drugs based interventions [26].

Numerous studies have demonstrated that Aβ induces oxidative stress in the brain primarily through activation of the multi-subunit enzyme complex NADPH oxidase 2 (NOX2) [4,22,27,28,29], which regulates microglial cell proliferation and stimulates cytokine release [30,31,32]. NOX2 is a major ROS source, producing superoxide anion radicals (O_2_^•−^). O_2_^•−^ rapidly convert into toxic oxidants like H_2_O_2_, hydroxyl radicals (^•^OH), hypochlorous acid (HOCl), nitrogen dioxide (^•^NO_2_), and peroxynitrite (ONOO^−^) that collectively drive oxidative stress [5,33]. Oxidative stress promotes misfolding and aggregation of both Aβ and tau protein [19,34,35,36]. These pathogenic forms of Aβ and tau protein further exacerbate oxidative stress through microglial activation [19,36,37], thereby creating a vicious cycle that reinforces neurodegeneration.

NOX2 inhibition is regarded as a promising strategy for developing targeted therapies for AD and other neurodegenerative disorders [4,38]. Among NOX2 inhibitors, GSK2795039—discovered by Hirano and colleagues [39]—has demonstrated significant specificity with favorable pharmacokinetic parameters both in vitro and in vivo, remaining the gold-standard NOX2 inhibitor [40]. GSK2795039 showed no cytotoxicity at concentrations effective for NOX2 inhibition and was well-tolerated in rodents, with no apparent side effects after 5 days of twice-daily administration [39]. GSK2795039 prevented acute oxidative stress induced by Aβ application to brain slices [22]. In vivo experiments demonstrated that two weeks of NOX2 inhibition via daily intracerebrovascular injections of GSK2795039 following oligomeric Aβ administration prevented the development of pathological behavioral changes in mice [22]. However, while NOX2 inhibition holds therapeutic potential, chronic systemic suppression may lead to adverse effects, such as attenuated immune responses, hyperinflammation, and increased risk of autoimmune disorders [41]. Together, these findings raise the question of whether chronic NOX2 inhibition is necessary or transient suppression of this enzyme is sufficient to disrupt the pathological cascade and prevent AD progression. Addressing this question is crucial when considering NOX2 as a potential therapeutic target.

In this study, we evaluated the long-term effects of transient NOX2 inhibition with GSK2795039, following intracerebroventricular Aβ injection in mice, at the physiological, cellular, and subcellular levels. To elucidate the specific contribution of microglial NOX2 to the observed effects, we investigated the efficacy of GSK2795039 against Aβ-induced toxicity in microglial cell culture.

## 2. Materials and Methods

### 2.1. In Vitro Assays

#### 2.1.1. Human Microglial Cell Culture

The human microglial clone 3 (HMC3) cell line (ATCC, cat. #CRL-3304) was cultured in minimum essential medium (MEM, Gibco, ThermoFisher Scientific, Waltham, MA, USA) supplemented with 10% FBS (Gibco, ThermoFisher Scientific, Waltham, MA, USA), 100 U/mL penicillin and 100 µg/mL streptomycin (Gibco, ThermoFisher Scientific, Waltham, MA, USA), 1% sodium pyruvate, and 1% GlutaMax (Gibco, ThermoFisher Scientific, Waltham, MA, USA) at 37 °C in a humidified atmosphere containing 5% CO_2_.

#### 2.1.2. Cell Treatment

A 10 mM stock of GSK2795039 (synthesized as described in [42]) was prepared in sterile DMSO (Sigma-Aldrich, St. Louis, MO, USA). Synthetic beta-amyloid peptide DAEFRHDSGYEVHHQKLVFFAEDVGSNKGAIIGLMVGGVVIA (Aβ, Peptide Specialty Laboratories GmbH, Heidelberg, Germany) was monomerised in 10% NH_4_OH, aliquoted and lyophilized. Commonly used doses of GSK2795039 (25 µM) and/or Aβ (10 µM) were used for cell treatment [42,43]. Equivalent DMSO volumes were added to the control and Aβ samples in all experiments. Each measurement was conducted in replicates (*n* = 3 per group), and results were averaged for statistical analysis.

#### 2.1.3. ELISA

HMC3 media were collected after 24 h incubation of the cells with Aβ and/or GSK2795039. The level of pro-inflammatory IL-6 was assessed using the IL-6 Human Uncoated ELISA Kit (Thermo Fisher Scientific, Waltham, MA, USA) in collected culture media.

Aβ level in HMC3 cells was measured using sandwich ELISA, as described elsewhere [44], after 24 h incubation with Aβ and GSK2795039.

#### 2.1.4. Real-Time PCR

HMC3 cells were seeded on a 24-well plate, incubated with Aβ and/or GSK2795039 for 24 h, and then frozen in liquid nitrogen. Total RNA was isolated from the frozen cells using ExtractRNA reagent (Evrogen, Moscow, Russia). Residual genomic DNA in the samples was removed by treatment with DNase I (Roche Diagnostics, Mannheim, Germany) for 30 min at 37 °C. RNA was isolated and purified using the CleanRNA standard kit (Evrogen, Moscow, Russia). RNA (2 μg) was reverse transcribed using the MMLV RT kit (Evrogen, Moscow, Russia). The reaction was performed using a BioRad thermal cycler at 40 °C for 45 min. The generated cDNAs were used as a template to determine mRNA expression level using 5X qPCRmix-HS SYBR (Evrogen, Moscow, Russia). The RT-PCR reaction was performed on a Roche PCR machine at 92 °C for 1 min followed by 60 × [92 °C 20 s, 61 °C 30 s, 72 °C 45 s] cycles. Primers for RT-PCR are listed in Table 1 (Evrogen, Moscow, Russia).

#### 2.1.5. Western Blot

After 24 h incubation with Aβ and GSK2795039, HMC3 cells were gently rinsed with PBS (Gibco, ThermoFisher Scientific, Waltham, MA, USA), frozen in liquid nitrogen, and lysed using RIPA lysis buffer (ThermoFisher Scientific, Waltham, MA, USA) containing protease inhibitors (Roche Diagnostics, Mannheim, Germany) on a rotator for 30 min at 4 °C. The lysates were centrifuged at 12,000× *g* for 10 min and the supernatant was collected. The samples were then separated on 10% SDS-PAGE and transferred to 0.22 μm nitrocellulose membrane (Bio-Rad, Hercules, CA, USA). Membranes were blocked in 5% non-fat milk in PBS, containing 0.1% Tween 20 (PBST) for 1 h at room temperature, followed by incubation with anti-NOX2 antibodies (1:1000, ThermoFisher Scientific, Waltham, MA, USA) overnight at 4 °C. After washing with PBST, the membrane was incubated with the appropriate HRP-conjugated anti-rabbit antibodies (1:5000, Santa Cruz Biotechnology, Dallas, Texas, USA) for 1 h at room temperature, washed again, and visualized with the substrate (SuperSignal™ West Femto Maximum Sensitivity Substrate kit, ThermoFisher Scientific, Waltham, MA, USA) using a Bio-Rad ChemiDoc MP instrument (Bio-Rad, Hercules, CA, USA). Densitometric analysis was performed using Image Lab 6.0.1 software (Bio-Rad, Hercules, CA, USA).

#### 2.1.6. Flow Cytometry Analysis

Fluorescent dyes: dihydrorhodamine 123 (7.5 μM, Ex/Em = 507/525 nm, ThermoFisher Scientific, Waltham, MA, USA), 1,1′,3,3,3′,3′-hexamethylindodicarbo-cyanine iodide (Ex/Em = 638/658 nm, ThermoFisher Scientific, Waltham, MA, USA), and monobromobimane (20 μM, Ex/Em = 395/495 nm, ThermoFisher Scientific, Waltham, MA, USA) were used to assess reactive oxygen species (ROS), mitochondrial membrane potential, and reduced glutathione (GSH) levels according to the manufacturer’s instructions. Stained cells were washed with Versene solution (Gibco, ThermoFisher Scientific, Waltham, MA, USA) and detached by TrypLE Express (Gibco, ThermoFisher Scientific, Waltham, MA, USA). MEM medium containing 10% FBS was added to all samples to inactivate the TrypLE enzyme. The percentage of dead cells in the population was estimated using propidium iodide (Ex/Em = 535/617 nm; Sigma, St. Louis, MO, USA). The dye was added to the cells at a concentration of 10 μg/mL for one minute prior to analysis on a flow cytometer. Propidium iodide positive cells were excluded from consideration when assessing ROS, GSH levels, and mitochondrial potential. Flow cytometry was performed using a BD LSR Fortessa flow cytometer (Becton Dickinson, Franklin Lakes, NJ, USA), and data analysis was performed using FlowJo software version 10.8.1 (Tree star, Ashland, OR, USA).

### 2.2. In Vivo Assays

#### 2.2.1. Preparation of Aβ Oligomers

Oligomeric Aβ (Sigma-Aldrich, St. Louis, MO, USA) was prepared as described elsewhere [22]. Aβ was dissolved in 0.2% NH_4_OH at 1 mg/mL and sonicated for 1 min. Prior to experiments, the solution was allowed to fibrilize for at least 1 h at 39 °C.

#### 2.2.2. Animal Model and Experimental Design

When selecting an experimental mouse model of AD, it was essential to determine the onset of pathology initiation; therefore, a sporadic model was chosen. Moreover, this model allowed us to avoid the effects of compensatory rearrangements in the tissues, which always accompany the development of pathology in transgenic models [45,46,47].

Experiments were performed on mature (3 month) male BALB/c mice (Laboratory Animal Nursery “Stolbovaya”, Russia). Mice were housed individually with food and water ad libitum. Animals were maintained on a 12 h light/dark cycle (lights on from 9 am to 9 pm) in a temperature-controlled room (22 °C ± 1 °C).

Mice were divided into three groups: 1. control (*n* = 5), 2. Aβ (*n* = 5), and 3. Aβ+GSK2795039 (*n* = 6). GSK2795039 (4.5 mg/mL), Aβ, or DMSO were administered in 1 μL. Doses of GSK2795039 and Aβ were previously justified [22]. Injections were made i.c.v. through the guide cannula (1 μL/min) to awake mice daily for 3 days. Experimental animals underwent neurosurgery under general anesthesia with isoflurane (Laboratorios Karizoo, Barcelona, Spain). Body temperature was maintained with a heating pad. Mice were placed in a stereotaxic apparatus and a guide cannula (stainless steel, 21 gauge) was implanted over the left lateral ventricle (AP = −0.7; L = 1.4; h = 2.2) according to the mouse brain atlas [48]. The site of cannulation was verified post mortem.

#### 2.2.3. Behavioral Tests

Behavioral tests were performed between 6 and 9 pm during the mice’s active phase. The EthoVision program (Noldus Information Technology, Wageningen, The Netherlands) was used for video recording and subsequent analysis.

The social behavior of the mice was assessed on day 13 using the “Social Interaction Test” described in the article [22]. This test was used on mice accustomed for 4 weeks to social isolation. All animals from one experimental group were placed in a round open field with a diameter of 60 cm for 1 h. Each mouse was marked with a colored spot on its back for identification. Aggressive behavior was quantified by the number of attacks, defined as throwing, jumping at a partner, and biting. The number of episodes and the total duration of fights were analyzed. Grouping was characterized as a prolonged (more than 4 s) friendly interaction involving three or more mice. The number and total time of episodes were assessed.

#### 2.2.4. Sample Collection

Mice were anesthetized with isoflurane and decapitated on day 14 after the first injection. The brain was quickly removed, one half of the brain (contralateral to the cannula) was analyzed by biochemical methods, and the other half was placed in 4% paraformaldehyde for subsequent immunohistochemical analysis.

#### 2.2.5. Biochemical Analysis

The half of the brain was immediately placed into 4 mL of an ice-cold isolation buffer containing 220 mM mannitol, 70 mM sucrose, 10 mM Hepes, 1 mM EGTA (pH 7.4) and homogenized manually with 30 strokes of tight-fitting pestle (Duran, Wheaton). The homogenate was centrifuged at 1600× *g* for 10 min at 4 °C. The pellet was resuspended in 3 mL of potassium phosphate buffer containing 125 mM KCl and 8 mM K_2_H_2_PO_4_ (pH 7.4) to obtain membrane-enriched fraction 1. The supernatant was further centrifuged at 12,000× *g* for 15 min at 4 °C. The cytosol-enriched supernatant constituted fraction 2. The resulting pellet was resuspended in 0.5 mL of isolation buffer without EGTA to obtain mitochondria-enriched fraction 3. All fractions were stored on ice during the experiment. The measurements were carried out using a multifunctional plate reader CLARIOstar Plus (BMGlabtech, Ortenberg, Germany).

The dynamics of ROS production was determined using Amplex Red (30 µM, Thermo Fisher Scientific). Measurements were performed with an excitation wavelength of 545 nm and emission wavelength of 600 nm for 30 min at 30 °C. The concentration of reduced thiol groups was determined by photometry using the Ellman method (1.3 mM 5,5′-dithiobis-2-nitrobenzoic acid (DTNB), ThermoFisher, USA). Absorbance measurements were performed at 415 nm using a calibration curve for cysteine. Peroxidized lipids were determined by fluorometry of reaction products with thiobarbituric acid (7.5 mM, Sigma-Aldrich, St. Louis, MO, USA). Fluorescence measurements were performed with an excitation wavelength of 530 nm and emission wavelength of 554 nm using a 1,1,3,3-tetraethoxypropane (Sigma-Aldrich, St. Louis, MO, USA) calibration curve. The protein content in fractions for normalization of biochemical parameters was determined using the Bradford method. Absorbance measurements were performed at 595 nm using a calibration curve for BSA (Dia-M, Moscow, Russia).

#### 2.2.6. Immunohistochemical Analysis

Brains were sectioned frontwise using a Leica VT 1200 S vibratome (Leica, Wetzlar, Germany). Free-floating slices (35 µm) were washed with PBS, containing Triton X-100 (0.3%), three times for 5 min, followed by blocking solution (BSA 1%, Triton X 0.3% in PBS) for 2 h. The slices were incubated overnight at 4 °C with primary anti-Iba1 antibody (1:1000; Wako, Japan) and then with the secondary anti-rabbit antibody (1:1000; Alexa Fluor 488, ThermoFisher, USA) for 2 h. After washout in PBS with 0.3% Triton X-100, the sections were mounted on gelatinized coverslips in Fluoromount medium (Sigma-Aldrich, USA). Immunostaining was analyzed using a Nikon E200 fluorescence microscope. For proper comparison, equivalent regions with similar proportions were selected for all groups. In each section, microglial cells in the CA1 and dentate gyrus (DG) areas were analyzed. Six sections per animal were used for averaging. Photomicrographs using 40X (0.25 numerical aperture) of stained fluorescence were quantified with the aid of ImageJ software version 1.52u (NIH, Bethesda, MD, USA). The number of Iba1^+^ cells and the cell area in 300 × 300 micron squares were calculated. The CA1 and DG areas of the hippocampus were used for analysis for the following reasons. The CA1 field is characterized by increased sensitivity to Aβ toxicity and changes in this area are a marker in the development of AD pathology. DG is an area of postnatal neurogenesis that is disrupted in patients with AD and in animals with an AD model [49]. In Alzheimer’s disease, microglia are involved in suppressing the normal process of neurogenesis, which makes the DG area the most valid for study.

### 2.3. Statistical Analysis

The statistical differences between the experimental groups were analyzed using one-way ANOVA with Tukey’s test, or Kruskal–Wallis multiple comparisons post hoc test, where appropriate. Data are presented as the mean values ± standard deviation (SD) or as box plots with min-max whiskers and Tukey quartile method; *p* ≤ 0.05 (*), *p* ≤ 0.01 (**), *p* ≤ 0.001 (***), *p* ≤ 0.0001 (****). The analysis was carried out using the GraphPad Prism software version 10.1.0 (GraphPad Software, San Diego, CA, USA).

## 3. Results

### 3.1. GSK2795039 Inhibits Oxidative Stress and Inflammatory Response to Aβ in Microglial Cells

**Figure 1:**
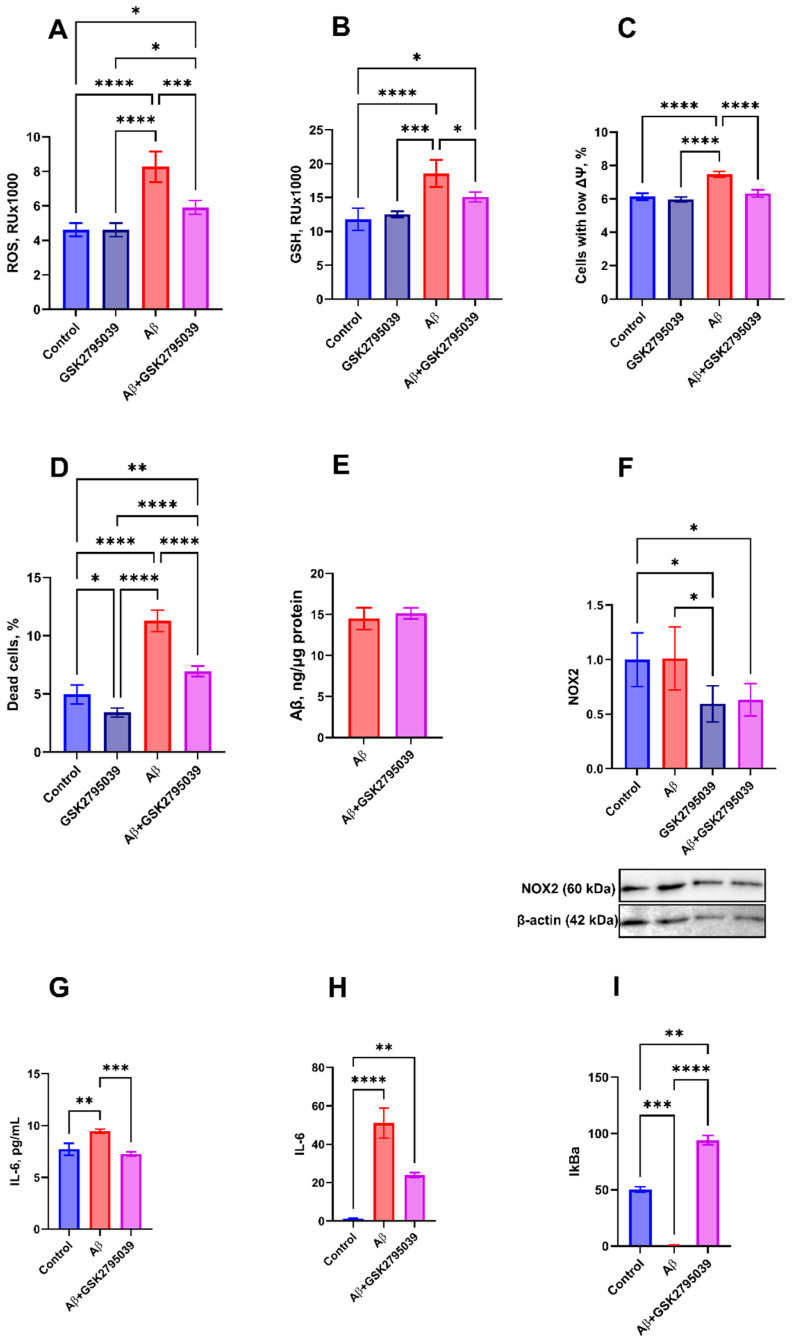
Effect of Aβ (10 µM) and GSK2795039 (25 µM) on HMC3 microglial cells after 24 h of incubation. Intracellular reactive oxygen species (ROS) (**A**) and reduced glutathione (GSH) (**B**) levels, percentage of cells with low mitochondrial membrane potential (ΔΨm) (**C**), and percentage of propidium iodide-positive (PI^+^, dead) cells (**D**) were assessed by flow cytometry. Effect of GSK2795039 on Aβ uptake by HMC3 cells was quantified by ELISA (**E**). NOX2 protein level in microglial cells was determined by Western blot (**F**). IL-6 secretion (supernatant concentration) was measured by ELISA (**G**). mRNA expression levels of IL-6 (**H**) and IkBa (**I**) were quantified by RT-qPCR. The statistical differences between the experimental groups were analyzed using one-way ANOVA with Tukey’s test. Data are presented as mean of three independent experiments ± SD, *n* = 3, *p* ≤ 0.05 (*), *p* ≤ 0.01 (**), *p* ≤ 0.001 (***), *p* ≤ 0.0001 (****).

Since NADPH oxidase 2 (NOX2) is the primary source of reactive oxygen species (ROS), we evaluated changes in redox parameters in HMC3 microglial cells after 24 h of incubation with Aβ and GSK2795039. Aβ significantly increased the levels of ROS and reduced glutathione (GSH), as well as the number of microglial cells with low mitochondrial potential. An increase in GSH level is characteristic of activated microglia, as these cells exhibit a distinct regulatory mechanism for GSH [50,51]. Addition of GSK2795039 did not change redox parameters of cells while effectively preventing Aβ-induced oxidative stress, restoring ROS, GSH levels, and the number of microglial cells with low mitochondrial potential nearly to control levels (Figure 1A–C). Under Aβ-induced oxidative stress, the percentage of propidium iodide-positive (PI^+^) dead cells in the population doubled, while the addition of GSK2795039 inhibited cell death (Figure 1D). Microglia cells internalized Aβ, and GSK2795039 did not affect the level of Aβ uptake by the cells (Figure 1E).

In addition to inhibiting NOX2 and associated oxidative stress, GSK2795039 reduced NOX2 level in microglial cells. Aβ did not significantly affect NOX2 level in HMC3 cells, whereas treatment with GSK2795039 decreased it by 40% (Figure 1F).

Changes in the redox status of microglia are accompanied by their activation and the development of inflammation [52]. To assess whether GSK2795039 could prevent Aβ-induced inflammation, we analyzed the levels of pro- and anti-inflammatory factors in HMC3 cells after 24 h of incubation with Aβ and GSK2795039.

Microglial activation under Aβ exposure was associated with increased expression and secretion of the pro-inflammatory cytokine IL-6 (Figure 1G,H). Additionally, inflammation development led to reduced expression of the anti-inflammatory factor IkBa, which inhibits the pro-inflammatory transcription factor NF-κB (Figure 1I). Treatment with GSK2795039 effectively suppressed Aβ-induced production of pro-inflammatory cytokines and significantly increased IkBa level in HMC3 cells.

### 3.2. GSK2795039 Prevents Aβ–Induced Behavioral Abnormalities in Mice

**Figure 2:**
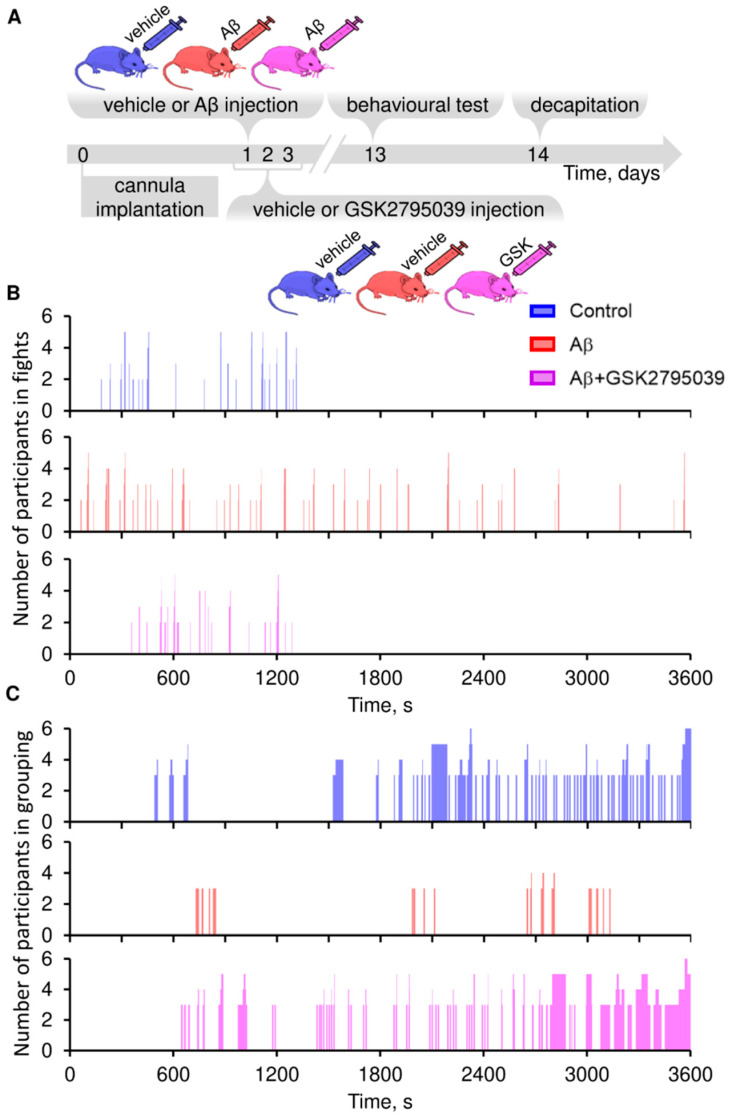
Effects of Aβ and GSK2795039 treatment on mice behavior. (**A**) Experimental timeline for in vivo studies (see Methods for details). (**B**,**C**) Social Interaction Test was performed on day 13 post-surgery. (**B**) Mice aggressive behavior was assessed on frequency and duration of fights (throwing, jumping at a partner, and biting), and (**C**) prosocial behavior on grouping (prolonged friendly interaction involving three or more mice). The duration of episodes and participant number are presented, *n* = 5.

To evaluate the effect of GSK2795039 on experimental animal behavior, we administered Aβ and GSK2795039 intracerebroventricularly (i.c.v.) and conducted behavioral studies according to the schematic (Figure 2A).

A comparative analysis of the behavior of mice from the control and Aβ groups using the “Social Interaction Test” showed a sharp increase in the number and duration of fights 13 days after amyloid administration (Figure 2B). Concurrently, the number of positive social interactions (grouping) decreased in the Aβ group (Figure 2C). The observed behavioral deviations were prevented by i.c.v. injections of GSK2795039.

### 3.3. GSK2795039 Reduces Oxidative Stress and Microglial Activation in the Brain Tissue

Effective suppression of behavioral deviations in mice by GSK2795039 suggested that they were caused by NOX2-mediated oxidative stress [22]. We assessed the levels of key biochemical markers of oxidative stress in the membrane, cytosolic, and mitochondrial fractions of brain homogenate, along with the potential for their reduction via GSK2795039.

**Figure 3:**
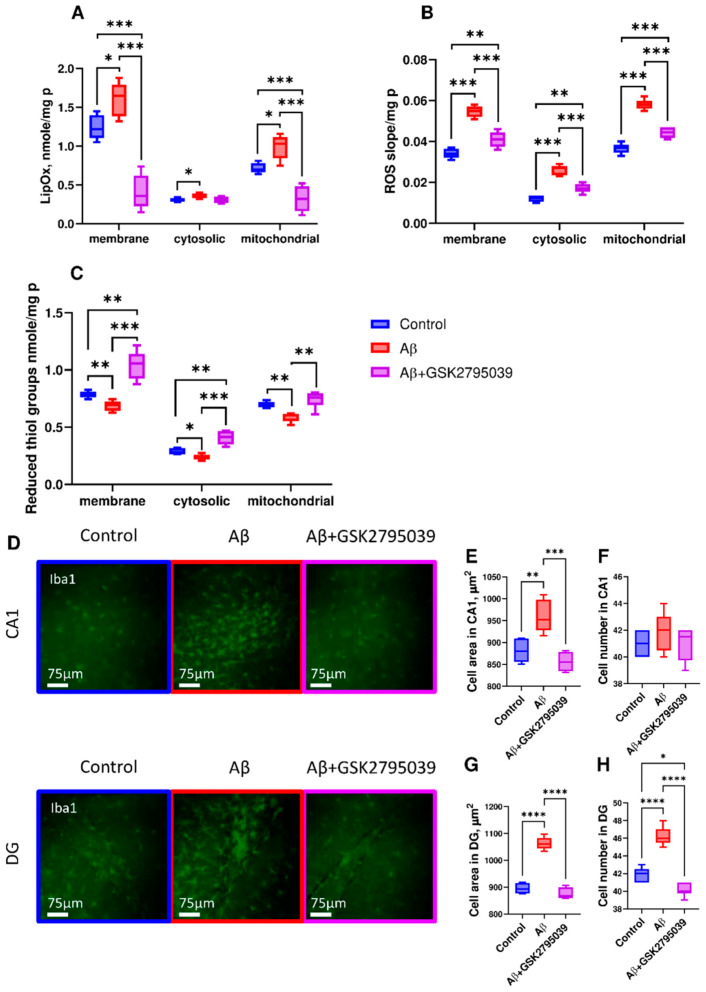
GSK2795039 attenuates Aβ-induced oxidative stress and microglial activation in mouse brain tissue. (**A**–**C**) Biochemical markers of oxidative stress in membrane, cytosolic, and mitochondrial fractions of brain homogenate: (**A**) lipid peroxidation (thiobarbituric acid-reactive substances, TBARS); (**B**) ROS production rate (Amplex Red assay, slope—Δfluorescence/min); (**C**) reduced thiol groups (Ellman’s method). Data was normalized on the total protein level in the fractions measured by Bradford protein assay. (**D**–**H**) Microglial activation in hippocampal CA1 and dentate gyrus (DG): (**D**) Representative Iba1+ immunofluorescence (scale: 75 µm); microglial area in CA1 (**E**) and DG (**G**) (µm^2^), and cell count in CA1 (**F**) and DG (**H**) (per 300 × 300 µm^2^ field). The statistical differences between the experimental groups were analyzed using Kruskal–Wallis multiple comparisons post hoc tests. Data are presented as box plots with min-max whiskers and Tukey quartile method, *n* = 5, *p* ≤ 0.05 (*), *p* ≤ 0.01 (**), *p* ≤ 0.001 (***), *p* ≤ 0.0001 (****).

Aβ increased lipid peroxidation, the rate of ROS production, and decreased the level of reduced thiol groups in brain tissues (Figure 3A–C). GSK2795039 prevented Aβ-induced changes in all three examined fractions, with the greatest efficacy in the membrane fraction. It should be noted that decreased level of reduced thiol groups in brain homogenate does not correlate with increased GSH level in microglial cells culture, apparently due to the fact that most of the homogenate consists of neuronal cells, which respond to Aβ by decreasing GSH level [53,54].

The development of oxidative stress was accompanied by changes in the number and morphology of microglia in the hippocampus of experimental animals (Figure 3D). Aβ increased the area occupied by microglial cells in the CA1 and DG regions (Figure 3D,E,G). The area of microglial cells in the DG region correlated with a significant increase in their number (Figure 3D,H). Treatment with GSK2795039 reduced the area occupied by microglial cells and their number to control levels in both hippocampal regions.

## 4. Discussion

The generation of free radicals by the NOX2 enzyme significantly contributes to the development of neuropathologies [55]. Inhibition of NOX2-induced oxidative stress is considered a promising therapeutic strategy for treating neurological disorders.

In 2015, a small-molecule NOX2 inhibitor, GSK2795039, was developed. GSK2795039 exhibited high selectivity and acted as a competitive NADPH oxidase inhibitor, suppressing NOX2-mediated ROS production and the consumption of NADPH and oxygen [39]. In subsequent years, GSK2795039 was successfully used to treat neurotrauma [32,56,57], intracranial hemorrhage [58], ischemic stroke [59], neuropathic pain [60], and Huntington’s disease in a cellular model [61]. Significant reductions in neuroinflammation, neuronal cell death, and oxidative stress were observed, along with GSH level in neural tissue and the recovery of long-term potentiation and synaptic transmission [56,57,60].

Oxidative stress and neuroinflammation are key pathogenic factors in AD [62]. Previously, we demonstrated that chronic administration of GSK2795039 for two weeks restored neuronal network activity and corrected behavioral abnormalities induced by Aβ in experimental animals [22]. However, studies in patients with X-linked chronic granulomatous disease have shown that systemic NOX2 inhibition itself can lead to adverse effects, such as increased infections and/or autoimmune disorders [38]. Therefore, the duration of NOX2 inhibition should be carefully controlled, and treatment should be limited to the minimally necessary period. The present study aimed to determine whether short-term NOX2 inhibition with GSK2795039 could prevent Aβ-induced inflammation and oxidative stress.

Short-term administration of GSK2795039, both in vitro and in vivo, effectively blocked Aβ-induced microglial activation. Treatment with GSK2795039 reduced microglial production of pro-inflammatory factors, changed microglial morphology in the hippocampus for homeostatic, and decreased the number of microglial cells in the dentate gyrus (Figure 1G–I and Figure 3D–H). Microglia play a dual role in AD pathogenesis. During early disease stages, microglia promote amyloid plaque formation and induce neuritic dystrophy, whereas in later stages they facilitate plaque compaction and reduce neuronal damage [63]. Inhibition of pathogenic microglial activation during the first three days following Aβ administration using GSK2795039 promoted transition of the cells to a homeostatic state (Figure 3D–H) without impairing their neuroprotective functions or Aβ clearance (Figure 1E).

Chronic microglial activation in AD leads to sustained production of pro-inflammatory factors, which in turn maintain microglia and astrocytes in an activated state, driving further cytokines and chemokines release [64]. This pathological cascade results in the chronic neuroinflammation that is observed in AD patients [65]. We showed that Aβ triggered elevated IL-6 production by microglial cells, while GSK2795039 suppressed their expression (Figure 1G–I). IL-6 contributes to memory impairment, glucose metabolism dysregulation, tau hyperphosphorylation, brain cell aging, and the production of other pro-inflammatory factors [66,67].

The transcription factor NF-κB plays a major role in AD-related neuroinflammation [68]. GSK2795039 restored the expression of the cytosolic inhibitor IκBα in microglia, which is downregulated by Aβ and suppresses NF-κB (Figure 1J). Previous studies have shown that targeting IκBα is an effective approach for reducing neuroinflammation in AD pathology [69].

GSK2795039 effectively suppressed neuroinflammation (Figure 1 and Figure 3D) and thus may mitigate brain cell aging, which, according to the inflammaging theory, is a direct consequence of inflammatory processes and further contributes to its progression. Low-grade inflammation is considered as a driver of age-related pathologies [67]. Consequently, short-term GSK2795039 treatment may not only suppress an already existing disease but also address its underlying causes.

Over the past decade, accumulating research data has revealed early brain changes during the development of AD-like pathology. AD patients have various neuropsychiatric symptoms, such as agitation, irritability, depression, anxiety, psychosis, and affective disturbances, long before cognitive decline [70]. Similarly, mice exhibit increased anxiety, aggression, and reduced prosocial behavior after 1–2 weeks of intracerebral Aβ injection [22]. In our study, we demonstrated that GSK2795039-mediated reduction in hippocampal microglial activation, numbers, and morphological changes correlated with decreased aggression during early pathology stages (Figure 2B,C). Since hippocampal microglial activation is linked to tau tangle accumulation and the severity of cognitive deficits, particularly in episodic memory, semantic memory, and perceptual speed [71], short-term GSK2795039 treatment may also restore memory and reduce tau aggregation in later stages.

Microglial activation and increased production of pro-inflammatory factors are tightly linked to oxidative stress and brain tissue damage, manifested as lipid peroxidation and generation of protein oxidation products that are toxic to neurons [72,73]. ROS induce various post-translational protein modifications. Reversible modifications (S-glutathionylation, sulfenylation, nitrosylation) can alter protein function but are restored upon redox status normalization, and irreversible post-translational modifications (sulfinylation, sulfonylation) target protein for degradation [74]. These modifications can directly affect the activity of redox-sensitive metabolic pathways [75,76]. Specifically, ROS inhibit critical glycolytic enzymes like glyceraldehyde-3-phosphate dehydrogenase (GAPDH) [77] and pyruvate kinase M2 (PKM2) [78], slowing glycolysis. Chronic glucose hypometabolism due to oxidative stress further exacerbates ROS production, since glucose serves as the sole fuel of the pentose phosphate pathway (PPP), the intracellular antioxidant system [69,70,71,72,73,74,75,76,77,78,79,80,81]. This creates a positive feedback loop between oxidative stress and glucose hypometabolism that drives neurodegenerative cascades in AD pathogenesis [4].

In the present study, analysis of various brain homogenate fractions and microglial cultures for oxidative stress markers demonstrated that GSK2795039 effectively prevented the shift of these markers toward pathological levels, thereby inhibiting the development of Aβ-induced chronic oxidative damage in brain tissue and microglial cells (Figure 3A–C). The reduction in NOX2-dependent oxidative stress by GSK2795039 involved not only enzyme inhibition [39] but also decreased NOX2 level in microglia (Figure 1F). The observed reduction in NOX2 protein level following GSK2795039 treatment likely results from enhanced degradation of the inactive enzyme. This is a common mechanism when an enzyme in combination with a ligand is removed by endocytosis from the cell surface and sent for degradation [82].

In conclusion, short-term NOX2 inhibition prevents the development of pathological changes including behavioral deficits, microglial activation, and oxidative stress during early Aβ pathology. Our findings highlight the crucial role of NOX2 in AD initiation and progression, and provide a foundation for developing novel therapeutic approaches. Targeting NOX2 with GSK2795039 may represent a promising strategy to counteract Aβ-induced toxicity in microglial cells. This approach could restore the balance between pro- and anti-inflammatory factors and ultimately reduce AD-associated neuroinflammation. At the same time, the question of the effectiveness of NOX2 inhibition at a late stage of pathology remains open. However, if the main negative effect on brain tissue is caused not by Aβ itself, but by Aβ-associated oxidative stress, then inhibition of NOX2 can also be effective in the late stages of pathology.

## Figures and Tables

**Table 1:** List of primers.

Target Gene	Sequence	NCBI Reference Sequence
IL-6-F	5′-GGAGCCCAGCTATGAACTCC-3′	NM_000600.5
IL-6-R	5′-GGTCAGGGGTGGTTATTGCA-3′	
IkBa-F	5′-GTGGGGCTGATGTCAACAGA-3′	NM_020529.3
IkBa-R	5′-GGTCAGTCACTCGAAGCACA-3′	
GAPDH-F	5′-TGCACCACCAACTGCTTAC-3′	NM_001256799.3
GAPDH-R	5′-GGCATGGACTGTGGTCATGAG-3′	

## Data Availability

The data presented in this study are available on request from the corresponding author.
